# Hints for the Treatment of Hydrophobia

**Published:** 1853-07

**Authors:** Marshall Hall


*Hints for the Treatment of Hydrophobia.* By Dr. Marshall Hall.—Many years ago I had the opportunity of watching the course of a case of hydrophobia. It occurred in a little boy; and I scarcely left the room during the eight and forty hours that he survived. But I need not detail the series of symptoms which occurred, and which I have described elsewhere, on the present occasion.

It has appeared to me that there are *three* modes of death in this disease :—1st. Sudden death from asphyxia. 2d. Sudden death from secondary asphyxia. 3d. Sudden death (for in all the cases I think the death is sudden and unexpected at the precise moment at which it occurs) from nervous exhaustion.

Either of these modes of dissolution would be averted by the timely institution of tracheotomy. Indeed, if this measure were adopted, the frightful seizures which occur from trying to take liquids would be obviated. These seizures consist in fearful attacks of laryngismus, and of convulsions of the neck and pharynx, but chiefly of laryngismus, with threatening of instant suffocation. These seizures would be disarmed of their force and terror by tracheotomy.

Tracheotomy thus obviating the *effects of laryngismus*—1st. The sudden death from asphyxia, the immediate result of asphyxia, could not occur!

There remains the sudden death from exhaustion. It is a question whether this would occur necessarily from the poison of hydrophobia. Why should it occur *necessarily* from this poison? No reason can be given for this; and we are not to be misled into a conclusion unsupported by facts, since, though all cases of hydrophobia have proved fatal, they have proved fatal by a mode by which they would not occur if tracheotomy were performed.

Could any measures be adopted to check the violence of the spasm,—laryngismus and its effects being obviated,—such as the hydrocyanic acid, and so to prevent the subsequent exhaustion? Or could any remedies be adopted to remove this exhaustion more directly, as wine or cinchona?

These hints I throw out for the consideration of my professional brethren, in the hope of good.—*Ibid.*

